# Protein–Protein Interactions in Virus–Host Systems

**DOI:** 10.3389/fmicb.2017.01557

**Published:** 2017-08-17

**Authors:** Anderson F. Brito, John W. Pinney

**Affiliations:** Department of Life Sciences, Centre for Integrative Systems Biology and Bioinformatics, Imperial College London London, United Kingdom

**Keywords:** PPI, virus-host interactions, protein interaction networks, molecular evolution, viral evolution, databases, structural biology, integrative biology

## Abstract

To study virus–host protein interactions, knowledge about viral and host protein architectures and repertoires, their particular evolutionary mechanisms, and information on relevant sources of biological data is essential. The purpose of this review article is to provide a thorough overview about these aspects. Protein domains are basic units defining protein interactions, and the uniqueness of viral domain repertoires, their mode of evolution, and their roles during viral infection make viruses interesting models of study. Mutations at protein interfaces can reduce or increase their binding affinities by changing protein electrostatics and structural properties. During the course of a viral infection, both pathogen and cellular proteins are constantly competing for binding partners. Endogenous interfaces mediating intraspecific interactions—viral–viral or host–host interactions—are constantly targeted and inhibited by exogenous interfaces mediating viral–host interactions. From a biomedical perspective, blocking such interactions is the main mechanism underlying antiviral therapies. Some proteins are able to bind multiple partners, and their modes of interaction define how fast these “hub proteins” evolve. “Party hubs” have multiple interfaces; they establish simultaneous/stable (domain–domain) interactions, and tend to evolve slowly. On the other hand, “date hubs” have few interfaces; they establish transient/weak (domain–motif) interactions by means of short linear peptides (15 or fewer residues), and can evolve faster. Viral infections are mediated by several protein–protein interactions (PPIs), which can be represented as networks (protein interaction networks, PINs), with proteins being depicted as nodes, and their interactions as edges. It has been suggested that viral proteins tend to establish interactions with more central and highly connected host proteins. In an evolutionary arms race, viral and host proteins are constantly changing their interface residues, either to evade or to optimize their binding capabilities. Apart from gaining and losing interactions via rewiring mechanisms, virus–host PINs also evolve via gene duplication (paralogy); conservation (orthology); horizontal gene transfer (HGT) (xenology); and molecular mimicry (convergence). The last sections of this review focus on PPI experimental approaches and their limitations, and provide an overview of sources of biomolecular data for studying virus–host protein interactions.

## Introduction

Compared to the relatively well-conserved processes found in cellular organisms, viruses demonstrate huge variations in terms of genomic composition, patterns of evolution, and protein function. While studying protein–protein interactions (PPIs) in virus–host systems, these variations on the pathogen side must be considered. A large proportion of the PPIs are mediated by domain–domain interactions (DDIs), and viruses belonging to different Baltimore groups have specific domain repertoires, providing different strategies and mechanisms of molecular recognition to accomplish their replication cycle (Zheng et al., 2014). In DDIs, molecular recognition is performed via amino acid residues located at interfaces of interaction. Under homeostatic conditions, host proteins interact with each other via (endogenous) interfaces that are also sometimes explored by viruses (exogenous interfaces), leading to competition for such molecular resources between viruses and hosts (Franzosa and Xia, 2011). Protein recognition events can occur as stable or transient interactions, and some proteins can establish interactions with multiple partners, either simultaneously (party hubs), or at different times (date hubs; Han et al., 2004). Such patterns of interaction can be studied in the context of the overall protein interaction network (PIN), in which each node shows particular properties (e.g., connectivity, centrality, etc.; Gursoy et al., 2008).

In terms of evolution, virus and host PPIs often evolve under a regime of arms race. In this phenomenon, one of the partners undergoes mutations that can in turn promote the fixation of mutations in its counterpart, causing both proteins to change over time in a way that retains their mutual recognition capabilities (Daugherty and Malik, 2012). Other common mechanisms of evolution in virus–host systems involve the acquisition of new proteins and interactions via gene duplication, horizontal gene transfer (HGT), and convergence (Alcami, 2003; Koonin et al., 2006; Garamszegi et al., 2013).

Protein interaction data are usually obtained using strategies such as, yeast two-hybrid (Y2H) and affinity-purification mass spectrometry (AP-MS), which present specific advantages and disadvantages (Gavin et al., 2006; Gingras et al., 2007). Different kinds of proteomic data are gathered in multiple independent databases, which provide researchers with information on protein classification, domains, interactions, GO terms, etc.

The purpose of this review is to give an overview on the main concepts required for studying protein interactions in virus–host systems. We also assess the availability of genomic, interaction, and structural data within several databases for all viral families described so far.

## Protein domains in the context of virus–host interactions

Domains are elementary protein structures that evolve independently from each other (Vogel et al., 2004). They have specific biological functions, and most proteins are composed of multiple domains (Apic et al., 2001). Since domains are the basic units by which proteins establish molecular interactions, PPIs can be better understood when they are seen from the level of DDIs (Lee et al., 2006; Yellaboina et al., 2011). Among different viruses, the domain repertoire varies according to the molecular structure of their genomes, which are the basis for their classification into seven viral groups (Baltimore, 1971; Table 1).

**Table 1:** Virus classification system (Baltimore groups).

**Group**	**Acronym**	**Members**
I	dsDNA	Double-stranded DNA viruses
II	ssDNA	Single-stranded DNA viruses
III	dsRNA	Double-stranded RNA viruses
IV	ssRNA(+)	Positive-sense single-stranded RNA viruses
V	ssRNA(-)	Negative-sense single-stranded RNA viruses
VI	ssRNA-RT	RNA reverse-transcribing viruses
VII	dsDNA-RT	DNA reverse-transcribing viruses

Viral domains observed among DNA viruses form groups I and II; RNA viruses from groups III, IV, and V; and retro transcribing viruses from groups VI and VII are strictly conserved within these groups, and each viral group uses a unique set of domains to carry out infections (Zheng et al., 2014).

Nearly two-thirds of the viral families are composed by viruses with small genomes of no more than 20,000 nt (see Supplementary Table 1). A direct consequence of this high level of genome compaction is that most viruses encode <30 proteins/domains (see Supplementary Table 1), which in turn are able to interact with multiple host targets and perform a large set of functions (Franzosa and Xia, 2011; Zheng et al., 2014). Another peculiarity of viral domains is their tendency to evolve by convergence, mimicking host interfaces and allowing their proteins to target and compete for host factors usually involved in crucial cellular processes (Franzosa and Xia, 2011; Daugherty and Malik, 2012). At the level of DDIs, RNA viruses tend to be better characterized in terms of the domains encoded and the functions performed. On the contrary, for some viruses with genomes larger than 20 kb (mostly group I DNA viruses, see Supplementary Table 1), information about domains is still limited, and many of their proteins have no assigned domain or known function (Zheng et al., 2014). Unfortunately, such scarcity of information currently limits the conclusions that can be drawn from such markedly incomplete protein data of large DNA viruses.

## Interfaces of protein interactions in virus–host systems

**Figure 1:**
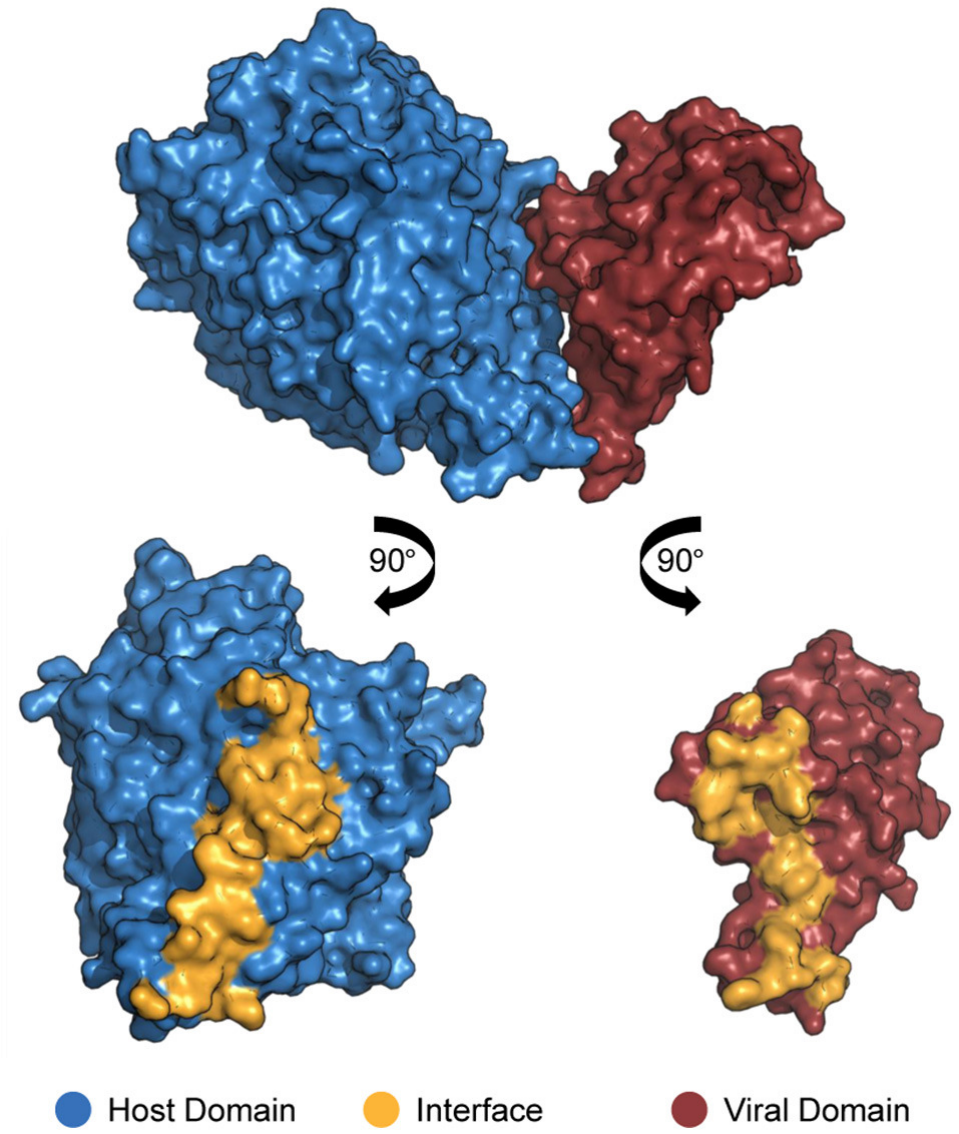
Structure of a DDI between a host domain (V-set domain, blue) and a viral domain (Herpes glycop D domain, red; PDB 3U82). By rotating each protein 90° outwards, the residues located at no more than 4.5 Å away from its partner's surface are colored yellow, indicating the interface residues.

In order for proteins to interact with each other, their respective binding sites must be in direct physical contact, either in a stable or transient mode (Byrum et al., 2012). Such binding sites are called “interfaces”: three-dimensional structures formed by sets of amino acid residues directly responsible for the recognition of binding partners (Figure 1; Franzosa and Xia, 2011). Deleterious or beneficial mutations occur especially on interfaces, affecting binding affinity due to impairment or improvement of protein electrostatic and structural properties (Daugherty and Malik, 2012).

In the context of virus–host PPIs, protein–binding sites can be classified as endogenous or exogenous interfaces. Endogenous interfaces are responsible for mediating interactions between proteins belonging to viral or host proteomes, i.e., host–host or virus–virus PPIs. On the other hand, exogenous interfaces mediate interactions between proteins belonging to distinct proteomes, as seen in virus–host PPIs (Franzosa and Xia, 2011).

In virus–host systems, extensive competition for interfaces is common between endogenous and exogenous partners, and viral proteins frequently interfere with host–host protein interactions (Franzosa et al., 2012). Such competition is so frequent that most of the proteins that have at least one known host–host interaction are also involved in virus–host interactions (Franzosa and Xia, 2011).

**Figure 2:**
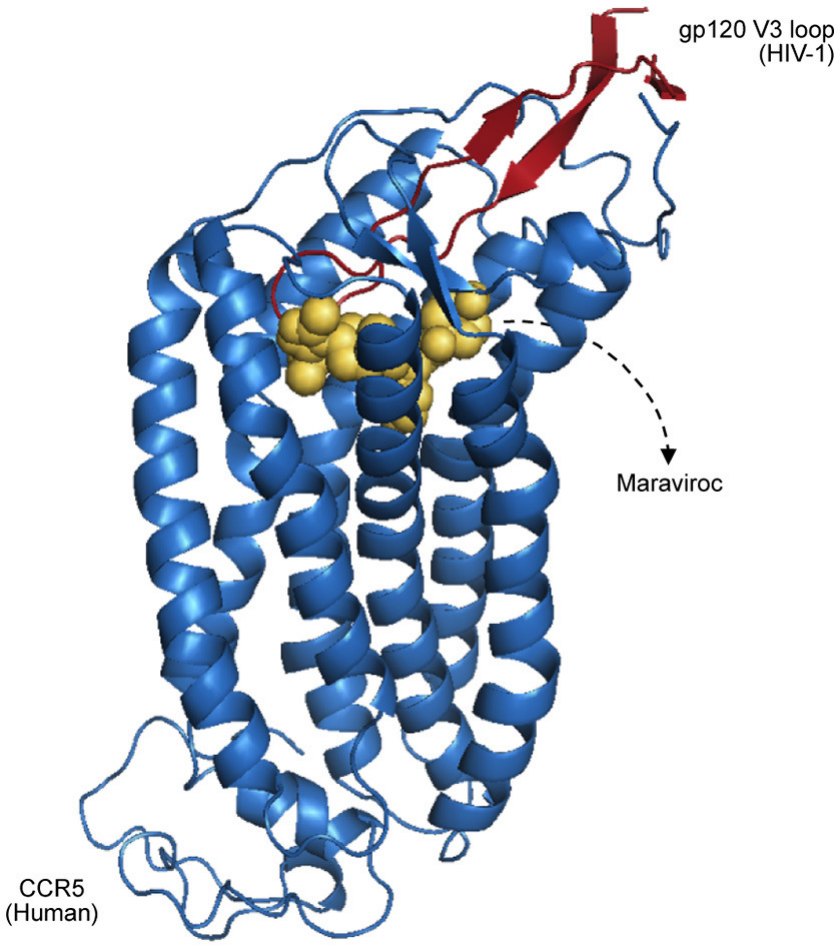
Representation of CCR5 (blue) bound with a Maraviroc molecule (yellow), superposed with the HIV-1 GP120 V3 loop (red), as proposed by Tamamis and Floudas (2014). As depicted, the drug occupies a CCR5 pocket, blocking its interaction with GP120.

Having a broader understanding of virus–host PPIs and their interfaces is crucial for the development of new antiviral therapies, like the design of small molecules capable of binding and blocking essential interactions of viral processes (Bailer and Haas, 2009; Gardner et al., 2015). A classic example of virus–host interactions being blocked at the interface level is the action of Maraviroc as an inhibitor of HIV-1 entry to host cells (PDB 4MBS). This drug binds the cellular co-receptor CCR5, preventing it from interacting with GP120 (Figure 2), an essential step of HIV-1 infection (Macarthur and Novak, 2008).

## Modes of protein interaction

PPIs commonly rely on large interfaces, whilst transient ones involve short linear peptides, as sequence motifs composed of 15 residues or less (Segura-Cabrera et al., 2013). Proteins that show a wide range of binding partners are called “hubs.” Hubs having only few interfaces are likely to interact transiently with different partners at different times (date hubs; Han et al., 2004), usually via domain–motif interactions (Franzosa and Xia, 2011). Conversely, proteins showing multiple interfaces tend to establish simultaneous interactions with multiple partners. Such proteins (party hubs) are likely to arrange themselves in complexes, via stable DDIs (Han et al., 2004).

Due to their mode of interaction and number of interface residues, party hubs tend to evolve slowly, as changes in their residues are likely to impair some interactions with specific partners (Fraser et al., 2002). The opposite scenario is observed among proteins that establish transient interactions, which usually evolve faster (Teichmann, 2002). Interestingly, most viral proteins interfering with cell signaling and regulatory pathways perform transient interactions with host proteins (Perkins et al., 2010), leading to severe changes in cellular metabolism (Segura-Cabrera et al., 2013). Unfortunately, compared to stable (domain–domain) interactions, transient (domain–motif) interactions are under-represented in PPI databases, mainly due to limitations associated with the methods used so far to obtain protein–protein interaction data (Russell et al., 2004).

## The evolution of protein interfaces and the virus–host arms race

In virus–host systems, interacting proteins are constantly losing and regaining their binding sites in order to evade or optimize interspecific PPIs. This process of constant change is known as an “evolutionary arms race” (Franzosa and Xia, 2011; Daugherty and Malik, 2012).

Under an arms race regime, proteins can evolve by offensive or defensive strategies. Host proteins evolve offensively when they are constantly changing as part of an effort to retain or restore their recognition capabilities to bind and neutralize viral factors, which in turn is under recurrent adaptation to evade the host's antagonist actions (Daugherty and Malik, 2012). For example, host immune system proteins in constant interaction with pathogen proteins frequently evolve by an offensive strategy. This scenario is usually found in mammals, whose antiviral proteins are under constant adaptation to recognize their antigens, showing a rapid mode of evolution (Lindblad-Toh et al., 2011). Conversely, defensive strategies are observed when host proteins targeted by viral antagonists undergo mutations to prevent pathogen proteins from binding their interfaces. As a response, this context can favor the fixation of novel mutations on viral interfaces, probably compensating for host evasion (Daugherty and Malik, 2012).

In this intricate virus–host arms race, endogenous and exogenous interfaces show different patterns of evolution. Host interfaces mediating host–host PPIs tend to be less variable than interfaces directly targeted by viral proteins (Franzosa and Xia, 2011). These proteins contain specific residues where small changes can drastically modify protein function and/or structure, and consequently their intraspecific binding affinity (Daugherty and Malik, 2012). This mode of evolution is especially observed in co-evolving host–host interfaces, where mutations can be potentially deleterious, and strong purifying selection acts to maintain the integrity of their binding sites (Franzosa and Xia, 2011; Daugherty and Malik, 2012). However, there are exceptions to this pattern. Taking into account that some endogenous binding sites overlap with exogenous interfaces (Franzosa and Xia, 2011), shared residues of endogenous interfaces can evolve faster due to competition with a viral antagonist (Elde and Malik, 2009).

## Protein interaction networks underlying viral processes

In order for pathogens to take over the cellular machinery and replicate themselves, molecular interactions must be established with their hosts. Such interactions are commonly represented using networks, where nodes represent proteins and edges connecting them denote direct physical interactions (Dyer et al., 2008; Bailer and Haas, 2009).

When a PIN is reconstructed, several properties of each protein can be calculated from its network topology, such as, connectivity (degree) and centrality (Gursoy et al., 2008). Some of these properties have been suggested to be biologically informative, although these findings are not strongly supported by data available so far (Mason and Verwoerd, 2007; Ratmann et al., 2009). As an example, it is well known that highly connected proteins (hubs) tend to interact with low-degree (non-hub) proteins instead of establishing interactions with other hubs (Maslov and Sneppen, 2002). Hub proteins are not always functionally essential, however, their high level of connectivity (degree) could evidence their involvement in multiple biological processes, in such a way that removing them from PINs can probably lead to negative pleiotropic effects (Ratmann et al., 2009).

In terms of virus–host interactions, it has been suggested that viral proteins tend to target more central and highly connected host proteins (Dyer et al., 2011; Zheng et al., 2014). Nonetheless, due to the overrepresentation of extensively studied proteins in PPI databases, assumptions drawn based on network properties could be mere sampling bias, and their meaning must be interpreted with caution (Ratmann et al., 2009; Dickerson et al., 2010). Another downside of host–pathogen PIN analysis is the incompleteness of their interactomes. The scarcity of interaction data in these systems has being a major analysis bottleneck, with most studies being either focused on extensively studied host–pathogen systems, or relying on transferring information by homology from such model systems to neglected ones (Ammari et al., 2016).

## How do protein interaction networks evolve?

Each viral family encodes a set of protein domains that are classified into several domain families based on their evolutionary relationships (Chothia et al., 2003). Large dsDNA viruses show the most variable protein domain repertoires, while most RNA and retrotranscribing viruses, due to their genome sizes, perform all their processes using few domains, which are reused throughout their entire infection cycles (Zheng et al., 2014). Owing to these proteomic peculiarities, viruses diversify and/or maintain their interaction capabilities via different mechanisms of molecular evolution, including conservation (orthology); HGT (xenology); gene duplication (paralogy); and molecular mimicry (convergence; Alcami, 2003; Koonin et al., 2006; Garamszegi et al., 2013).

**Figure 3:**
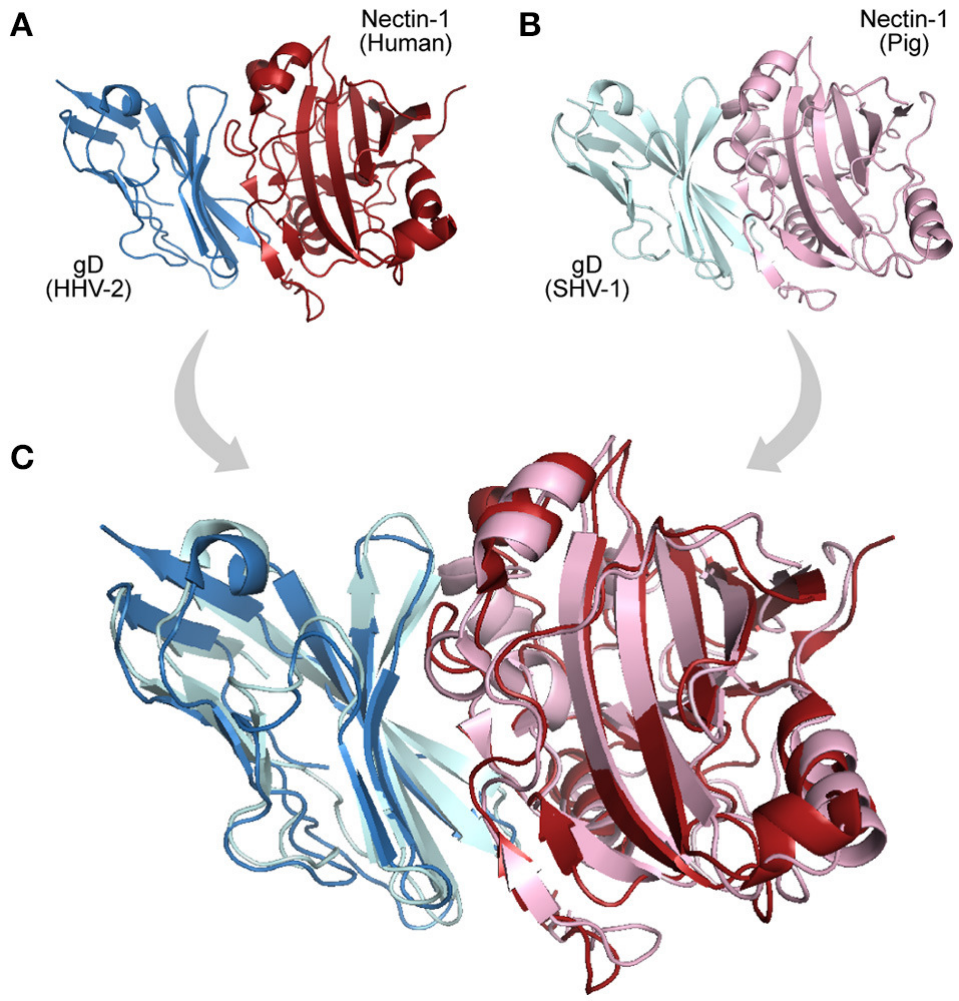
Interologs: homologous interactions. **(A)** Protein-protein interaction (PDB 4MYW) between a human Nectin-1 (blue protein) and a Glycoprotein D encoded by a Human Herpesvirus 2 (gD, red protein). **(B)** Interaction (PDB 5X5W) between swine Nectin-1 (light cyan protein) and Suid Herpesvirus 1 gD (pink protein). **(C)** Superposition of the interologs: both PPIs are found in distinct but homologous systems.

In the mode of evolution by orthology, if a domain pair known to interact is found in two closely related systems (organisms), these domains are likely to be real interacting partners in both systems (interologs). Although the mere presence of orthologs in two systems does not necessarily imply a direct interaction among them (Riley et al., 2005), their co-occurrence could be indicative of potentially conserved interactions, making these proteins good targets for further studies (Dyer et al., 2011). Examples of interologs are observed among herpesviruses, DNA viruses that show a large set of genes and interactions shared among almost all members of the family Herpesviridae (Bailer and Haas, 2009). An example of such conservation in observed in the interaction between the cellular receptor Nectin-1 and the envelope glycoprotein D encoded by alphaherpesviruses. Nectins are commonly found at adherens junction (GO:0005912), and are used by viruses as cell entry mediators (GO:0046718). Figure 3 illustrates such an interaction in two host–virus pairs: “human × HHV-2,” and “pig × SHV-1.”

**Figure 4:**
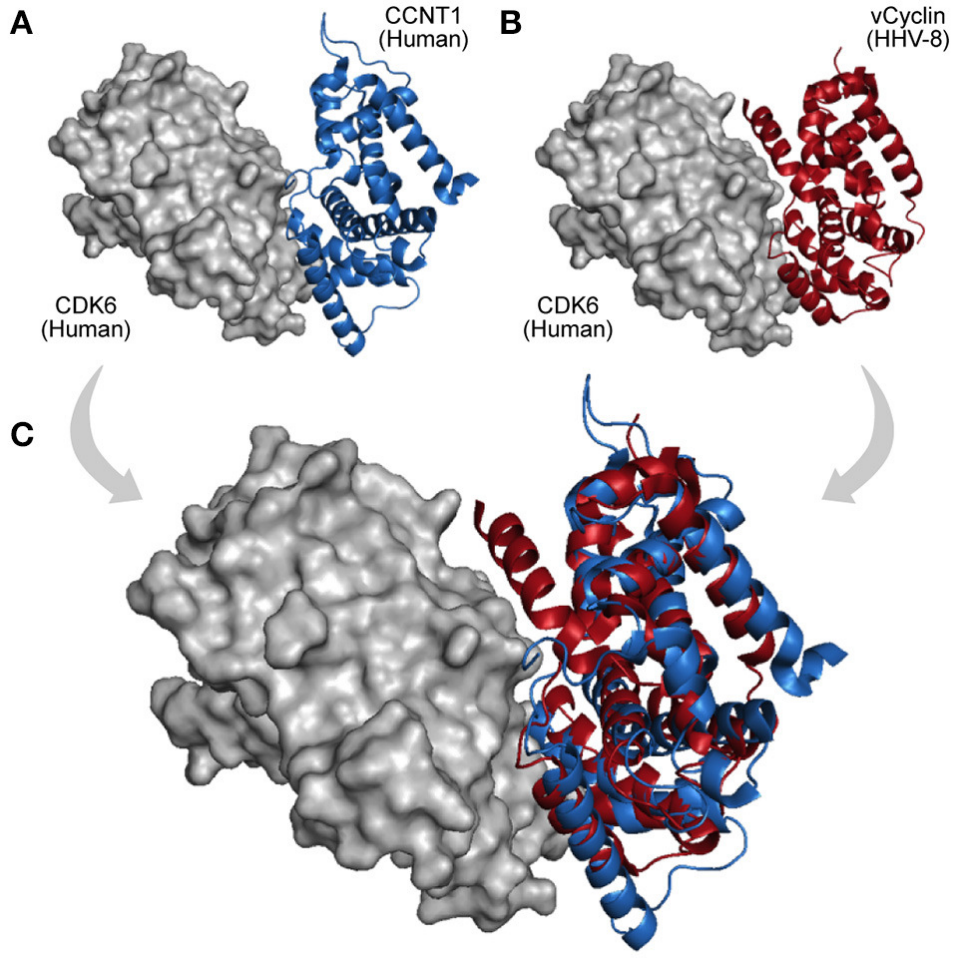
A viral PPI interaction derived from HGT. **(A)** In host protein networks, CDK6 (gray protein) originally establishes interaction with human Cyclin-A/CCNT1 (blue protein, PDB 3MI9). **(B)** Interestingly, a viral cyclin encoded by HHV-8, probably acquired by HGT (red protein, PDB 1G3N), is also able to establish similar interactions. **(C)** As both proteins share the same domain (Cyclin_N; PF00134), the structural superposition between the human cyclin **(A)** and its viral cognate **(B)** reveals their folding and binding similarities.

HGT is a process of genome recombination by means of which some viruses acquire one or more genes from non-parental organisms, a mechanism of evolution especially observed among large DNA viruses, which usually acquire new genes from other viruses, bacteria, or from their hosts (Shackelton and Holmes, 2004). Once a viral genome has incorporated a new gene, the protein product can be optimized and integrated into its virus–host network (Daugherty and Malik, 2012). Large dsDNA viruses, such as, poxviruses and herpesviruses, have been shown to be remarkably prone to acquire and domesticate exogenous genes within several functional categories (Raftery et al., 2000; Hughes and Friedman, 2005). Figure 4B shows an interaction between a human CDK6 and a Cyclin encoded by the *Human Herpesvirus 8*. This interaction is part of an immune system process (GO:0006955), and takes place in the extracellular region (GO:0005576). The viral cyclin (vCyclin) was probably acquired by HGT, and is capable of modulating cellular growth (GO:0005125) in similar ways to cellular cyclins D (Figure 4; Godden-Kent et al., 1997).

Gene duplication (paralogy) is another usual mechanism of protein network evolution, and duplicated genes are common in some viral genomes. After a duplication event, each paralog can undergo independent mutations, giving rise to new biological functions (Barabasi and Oltvai, 2004; Ratmann et al., 2009). Similarly to HGT events, gene duplications are evolutionary processes mainly found among dsDNA viruses, as observed in herpesviruses, adenoviruses and poxviruses (Shackelton and Holmes, 2004). An example of evolution by gene duplication is observed for the herpesvirus Glycoprotein D previously shown in Figure 1. Some alphaherpesviruses express a second copy of that protein, glycoprotein G (gG), a paralog that does not act as a viral entry mediators, but in fact shows a modified function, binding a broad range of chemokines to prevent their interaction with specific cellular receptors (Bryant et al., 2003). Interestingly, the presence of paralogs in PINs is not exclusive to viruses.

**Figure 5:**
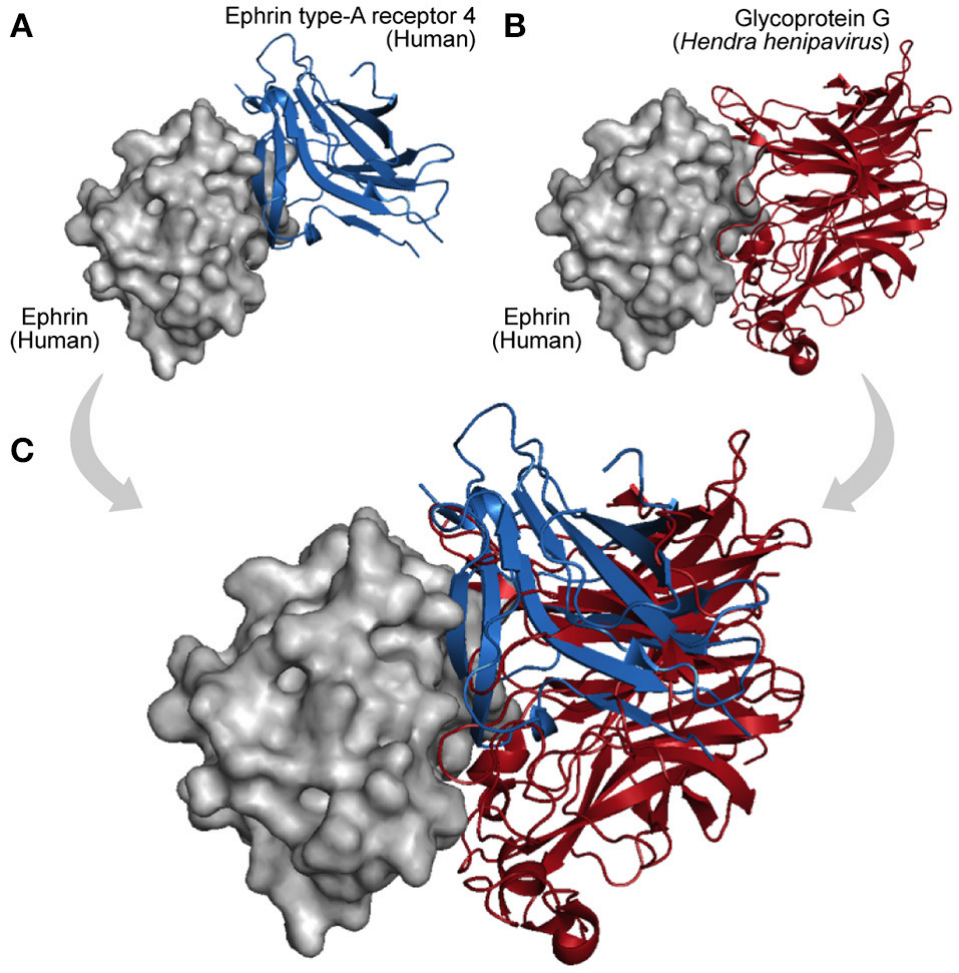
Interaction convergence. **(A)** In physiological conditions the cell surface Ephrin (gray protein) binds its Ephrin type-A receptor 4 (blue protein, PDB 3GXU). **(B)** However, during infections of the Paramixovirus *Hendra henipavirus*, the Ephrin interface is also used by the viral Glycoprotein G, which by convergence evolved its binding capacity (red protein, PDB 2VSK). **(C)** As shown in the superposition, Ephrin type-A receptor 4 and the viral Glycoprotein G can compete for the same interface on the Ephrin surface.

Finally, acquisition of a new interaction partner via convergent evolution is also a recurrent mechanism in virus–host networks. As they evolve at faster mutation rates, viruses can rapidly acquire new binding partners by mimicking and targeting interfaces of host proteins (Elde and Malik, 2009; Standfuss, 2015). A particular example is observed among *Dengue* viruses, *Vaccinia* viruses, and HIV-1, which independently acquired similar mechanisms of protein interaction and RNA recognition, which are essential to promote genome replication and mRNA translation (Garcia-Montalvo et al., 2004; Alvarez et al., 2006; Katsafanas and Moss, 2007). In this way, viruses can evolve not only by homology (HGT, duplication, and conservation) but also by analogy, allowing them to share interacting partners and adopt common strategies of infection (Dyer et al., 2008; Bailer and Haas, 2009; Segura-Cabrera et al., 2013). Figure 5 shows an example of convergent evolution. The human Ephrin-B2 is a cell surface transmembrane ligand of Ephrin receptors (Figure 5A; Qin et al., 2010), and the Glycoprotein G encoded by the Paramixovirus *Hendra henipavirus* is an envelope component (GO:0019031) that mimics this interaction with Ephrin receptors using an interface similar to the one explored by Ephrin type-A receptors 4 (Figure 5B).

## Protein interaction data: experimental approaches and limitations

Among the experimental techniques applied to identify virus–host protein interactions, Y2H and AP-MS are the most extensively used, together contributing to more than 90% of the information available in public databases (Guirimand et al., 2014), with the remaining data being obtained by GST-pull-down, luminescence, protease assay, surface plasmon resonance (SPR), and other techniques.

Y2H is efficient at detecting weak/transient domain–motif interactions (Gavin et al., 2006), however, one drawback is that it does not provide precise information about the domains involved in the interactions (Riley et al., 2005; Lee et al., 2006; Segura-Cabrera et al., 2013). Another disadvantage of Y2H screens is that PPI detection takes place within the nucleus. As some proteins are not naturally found in this cellular compartment, they are usually not identified as interactors, resulting in a bias that increases the proportion of false negatives (Von Mering et al., 2002). Additionally, a large number of entries in some databases describe binary interactions that are not physiologically feasible, i.e., even though two proteins are biochemically able to interact, if they do not share the same temporal and spatial compartment in a given biological process, their physical contact would not happen in natural conditions (Russell and Aloy, 2008).

AP-MS works in a different way. In these assays, proteins of interest (baits) are tagged with a recombinant fusion tag, which is then used to purify baits and their respective interacting partners (preys). Once purified, each component in the protein complex can be determined by mass spectrometry (Von Mering et al., 2002; Gingras et al., 2007). As a consequence, this technique is more efficient at identifying stable interactions among proteins of the same functional category (Gavin et al., 2006; Chiang et al., 2007). On the other hand, there are some disadvantages associated with affinity purification assays. An intrinsic issue is the use of tags by itself. By tagging the target proteins, their folding can be affected, preventing their normal functioning and raising the level of false positives (Bauer and Kuster, 2003; Meyer and Selbach, 2015). Another downside of this method is that reactions occur in ectopic environments, in other words, outside the normal physiological context of the PPIs. Additionally, for detecting interactions, affinity purification assays require proteins to be overexpressed, which can give rise to artifactual results (Bauer and Kuster, 2003).

To tackle these problems, methods based on Förster Resonance Energy Transfer (FRET) have increasingly gaining popularity. FRET-Based techniques allow the expression of each respective target protein in their native environment, as a single molecule fused with a fluorescent donor/acceptor. Such methods rely on a physical phenomenon of energy transferring between two fluorophores (in this context, interacting proteins): one protein receives light at a specific wavelength and transfers the energy to its interactor, which in turn emits light of other wavelength/color, which is captured by sensors (Xing et al., 2016). Although still an expensive alternative, FRET-based methods could become a suitable solution for detecting dynamic PPI in viral infections (Pfleger and Eidne, 2006; Xing et al., 2016).

So far, the main problem associated with PPI data is the poor overlap among datasets, as large proportions of the interactions are not shared among different experimental screens (Ratmann et al., 2009). This implies that although current PPI assays are high-throughput, their levels of completeness are low, leading to incomplete coverage of the interaction space (Von Mering et al., 2002). This is especially observed in Y2H assays, for which sensitivity (true positive rate) is low, ranging from 20 to 40%, implying that most pairwise interactions are not identified, falling in the false negative space (Bailer and Haas, 2009; Braun et al., 2009).

Another problem is that many databases are constructed by integrating data derived from several low throughput studies, which leads to some proteins being overrepresented in PINs (Bailer and Haas, 2009). It occurs mainly due to the bias associated with highly studied genes, such as, *p53* and other genes often related to human diseases (Sinen and Koyutürk, 2010). Proteins encoded by these genes usually emerge as highly connected nodes in the PIN, which may lead to erroneous conclusions, especially when it comes to applying the available data as input for predictive models (Riley et al., 2005; Bailer and Haas, 2009).

However, although high confidence data are still scarce, the virus–host PPI data available so far allow us to conduct large-scale comparative studies to understand the fundamental cellular functions targeted by viruses from different viral families, as well as the evolution of virus–host PINs (Navratil et al., 2009).

## Sources of biomolecular data for virus–host interaction studies

Integrative approaches are essential for the deep understanding of the evolutionary aspects of virus–host PINs. The data types applied for this purpose comes from different biological dimensions: protein sequence, domain composition, PPIs, DDIs, gene ontology terms, and taxonomic data, and several databases providing such biological information are available (see Table 2).

**Table 2:** Sources of biomolecular data for studies on virus-host interactions.

**Data types^†^ & Databases**	**Protein classification**	**Protein sequence**	**Protein structure**	**Protein Interaction**	**Domain interaction**	**Genomic sequence**	**GO terms**	**PSICQUIC member?**	Data source^*^	**References**
3DID			∘	•	•		∘	No	[I]	Mosca et al., 2014
BIND	∘			•				Yes	[E]	Bader et al., 2003
BIOGRID				•				Yes	[E] [T]	Chatr-Aryamontri et al., 2015
DIP	∘	∘		•			∘	Yes	[E]	Xenarios et al., 2002
GENERIF				•				No	[E] [T]	Mitchell et al., 2003
HIV-1 Human Protein Interaction		∘		•		•		No	[T]	Ptak et al., 2008
HPIDb				•				Yes	[I] [P]	Kumar and Nanduri, 2010
HPRD	∘	∘	∘	•			∘	Yes	[E]	Prasad et al., 2009
InnateDB				•			∘	Yes	[E] [T]	Lynn et al., 2008
IntAct	•	∘		•			∘	Yes	[E] [T]	Aranda et al., 2010
InterPro	•	∘	∘	∘			∘	–	[E] [I]	Mitchell et al., 2015
iPfam	•		∘	•	•			No	[I]	Finn et al., 2014b
MatrixDB	∘	∘	∘	•			∘	Yes	[E] [P]	Launay et al., 2015
MINT	∘	∘	∘	•			∘	Yes	[E] [T]	Licata et al., 2012
MPPI				•				No	[T]	Pagel et al., 2005
Negatome				•	•			No	[P] [T]	Blohm et al., 2014
NetworKIN				•				No	[P]	Linding et al., 2008
Pfam	•	•	∘	•	•		∘	No	[I] [P]	Finn et al., 2014a
PDB	∘	•	•	•	•		∘	No	[E]	Berman et al., 2000
PQS/PISA			•	•	•			No	[A] [I]	Henrick and Thornton, 1998
Reactome	•			•			∘	Yes	[I] [T]	Joshi-Tope et al., 2005
SCOP	•	•	∘					–	[A] [I]	Andreeva et al., 2014
Superfamily	•	∘				∘	∘	–	[A] [P]	Gough and Chothia, 2002
UniProt	∘	•	∘	∘		•	∘	Yes	[I]	UniProt Consortium, 2014
Viral Genomes NCBI		∘				•		–	[I]	Brister et al., 2015
VirHostNet				•				Yes	[I] [T]	Guirimand et al., 2014
VirusMentha				•				No	[I] [T]	Calderone et al., 2015

* *A, Annotation; E, Experimental data; I, Integration of multiple databases; P, Prediction; T, Text mining*.

† *•, primary data type; °, data retrieved from links to secondary sources*.

SCOP (Andreeva et al., 2014), Superfamily (Gough and Chothia, 2002), and Pfam (Finn et al., 2014a) provide valuable information on protein and domain classifications, such as, domain organizations, functional annotations, and taxonomic distributions of proteins encoded by completely sequenced genomes. To explore genetic diversity, databases such as (UniProt Consortium, 2014) and Genbank/NCBI (Benson et al., 2014) offer a variety of genome and protein sequences from viruses and their hosts. Protein structure data, such as, protein interfaces and chemical properties, can be found on PQS/PISA (Henrick and Thornton, 1998) and especially on PDB (Berman et al., 2000). Supplementary Table 2 includes a list of 1,100 virus–host PPIs with solved PDB structures, which are classified by viral/host taxonomy, GO terms, protein domains, among other biological information.

Viral and host PPI data can be retrieved from multiple databases, but VirHostNet (Guirimand et al., 2014) and VirusMentha (Calderone et al., 2015) are databases entirely dedicated to virus–host interactions. Most of their entries come from external and semi-independent sources, such as, MINT (Licata et al., 2012), IntAct (Aranda et al., 2010), DIP (Xenarios et al., 2002), and BIOGRID (Chatr-Aryamontri et al., 2015), which predominantly store experimental and literature-derived data. Hence, these datasets show slightly different sets of binary interactions, which cover different parts of the interaction space (Ratmann et al., 2009). To circumvent these problems, an integrative platform was proposed as a solution to integrate all entries, building a single, comprehensive, and non-redundant database. The Proteomics Standard Initiative developed the Common Query Interface (PSICQUIC), a platform to retrieve molecular interaction data from multiple databases storing binary interactions in PSI-MI format (Aranda et al., 2011).

Finally, most of the entries of the aforementioned databases are usually associated with external gene ontology (GO) information (Ashburner et al., 2000), such as, biological processes, molecular functions, and cellular compartments where interactions are likely to occur. Altogether, functional, evolutionary and interaction data make it possible to integrate all knowledge accumulated so far, and construct predictive models for virus–host interactions.

**Figure 6:**
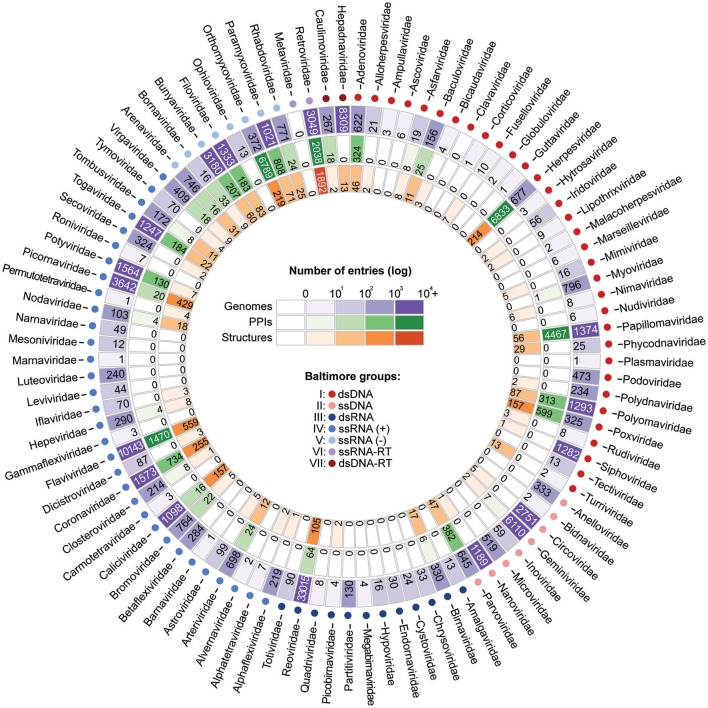
Current scenario of data availability for studying viral protein interactions. The outer ring (purple) shows the number of species-specific whole genomes sequenced so far. Such data provides us valuable information on genetic diversity. The PPI data (green ring) provide binary information about pairs of interacting proteins. Finally, the inner ring (orange) presents the structural data available, which allow the investigation of PPIs at the atomic level. As shown, for some viral families substantial amounts of data are available at all three levels.

For those interested in studying the evolution of viral PPIs by means of integrative approaches, three types of information are of particular interest: sequence diversity, binary protein interactions, and protein structures. By collecting viral biomolecular data from NCBI (Viral Genomes), VirHostNet (PPIs), and PDB (Structures) it is possible to assess the level of data availability for several viral families. As shown in Figure 6, most families still have only a limited amount of data, far from faithfully representing the true complexity of a virus protein interaction network (Dyer et al., 2011). However, the amount of data available for at least 15 families has shown to be favorable for evolutionary studies on viral PPIs. Among the viral families with consistent data availability there are five dsDNA families (Adenoviridae, Herpesviridae, Papillomaviridae, Polyomaviridae, Poxviridae); one ssDNA (Parvoviridae); one dsRNA (Reoviridae); three ssRNA(+) (Coronaviridae, Flaviviridae, Picornaviridae); four ssRNA(-) (Bunyaviridae, Filoviridae, Orthomyxoviridae, Paramyxoviridae), and one ssRNA-RT (Retroviridae; Figure 6). As previously mentioned, these distinct viral groups evolve under particular genetic mechanisms, and are good models for understanding the evolution of PPIS at the molecular and network levels.

## Conclusion

Viruses are pathogens with rather compact genomes that nevertheless provide them with versatile molecular tools able to cause extensive changes in cellular processes. Such versatility can be credited to domain repertoires encoded by viruses, as well as to their mechanisms of molecular evolution. The aspects addressed in this review provide starting points not just to virologists willing to explore integrative approaches to understand viral evolution, but also to computational biologists wanting to understand more about the peculiarities of viral biology in order to develop predictive models for virus–host PPIs.

Advances in biomolecular research over the last decades now allow us to tackle important questions regarding virus–host interactions by integrating data from multiple levels of biological complexity. As shown in Figure 6, for at least 15 viral families, large amounts of information on sequence diversity, protein interactions, and structures are available, allowing us to better understand how viruses evolve their mechanisms of interaction alongside their hosts. Additional studies on such evolutionary aspects could help us to develop new strategies for PPI inhibition and provide us with extra knowledge to explain cases of host switch, as well as the expansion of pathogen virulence and host range in emerging viral diseases.

## Author contributions

AB and JP wrote the manuscript. AB collected the data and created the figures. All authors read and approved the final manuscript.

### Conflict of interest statement

The authors declare that the research was conducted in the absence of any commercial or financial relationships that could be construed as a potential conflict of interest.
