# Supplementary material for: Protein–Protein Interactions in Virus–Host Systems
Source: Front Microbiol. 2017 Aug 17;8:1557. doi: 10.3389/fmicb.2017.01557 (PMC5562681; doi:10.3389/fmicb.2017.01557)
Supplement: Supplementary file 1 [file Table1.PDF]

Supplementary Table 1. Genomic features of viruses from different families.

Class		Group	Viral family	Genomic Segments	Minimum/Maximum Genome Size (bp)		Minimum/Maximum Proteins encoded	
●	1	dsDNA	Adenoviridae	1-2	26163	70137	15	77
●	1	dsDNA	Alloherpesviridae	1	134226	295146	90	163
●	1	dsDNA	Ampullaviridae	1	23814	23814	57	57
●	1	dsDNA	Ascoviridae	1	119343	186262	123	180
●	1	dsDNA	Asfarviridae	1	170101	170101	159	159
●	1	dsDNA	Baculoviridae	1	81755	178733	89	183
●	1	dsDNA	Bicaudaviridae	1	62730	76107	72	75
●	1	dsDNA	Clavaviridae	NA	NA	NA	NA	NA
●	1	dsDNA	Corticoviridae	1	10079	10079	22	22
●	1	dsDNA	Fuselloviridae	1	14796	24186	24	38
●	1	dsDNA	Globuloviridae	1	20933	28337	38	48
●	1	dsDNA	Guttaviridae	NA	NA	NA	NA	NA
●	1	dsDNA	Herpesviridae	1	108409	241087	69	274
●	1	dsDNA	Hytrosaviridae	1	124279	190032	108	160
●	1	dsDNA	Iridoviridae	1	102653	220222	95	468
●	1	dsDNA	Lipothrixviridae	1	20869	41172	40	73
●	1	dsDNA	Malacoherpesviridae	1	207439	207439	127	127
●	1	dsDNA	Marseilleviridae	1	346754	369360	403	444
●	1	dsDNA	Mimiviridae	1	617453	1259197	544	1176
●	1	dsDNA	Myoviridae	1	11624	497513	14	675
●	1	dsDNA	Nimaviridae	1	305108	305108	532	532
●	1	dsDNA	Nudiviridae	1	96944	231621	98	139
●	1	dsDNA	Papillomaviridae	1	7020	8607	3	15
●	1	dsDNA	Phycodnaviridae	1	154641	459984	150	886
●	1	dsDNA	Plasmaviridae	1	11965	11965	16	16
●	1	dsDNA	Podoviridae	1	11660	145865	11	198
●	1	dsDNA	Polydnaviridae	15-105	185221	567670	5	155
●	1	dsDNA	Polyomaviridae	1	3962	7369	4	9
●	1	dsDNA	Poxviridae	1	134431	359853	130	334
●	1	dsDNA	Rudiviridae	1	24655	35450	37	54
●	1	dsDNA	Siphoviridae	1	14270	279967	20	448
●	1	dsDNA	Tectiviridae	1	14319	14935	27	32
●	1	dsDNA	Turriviridae	1	16622	17663	34	36
●	2	ssDNA	Anelloviridae	1	2064	3899	1	6
●	2	ssDNA	Bidnaviridae	2	12565	12565	7	7
●	2	ssDNA	Circoviridae	1-2	859	4715	1	5
●	2	ssDNA	Geminiviridae	1-3	2383	6518	1	12
●	2	ssDNA	Inoviridae	1-3	4491	36108	3	44
●	2	ssDNA	Microviridae	1	4129	6478	6	13
●	2	ssDNA	Nanoviridae	6-8	6396	8091	5	9
●	2	ssDNA	Parvoviridae	1	3776	6321	2	9
●	3	dsRNA	Amalgaviridae	1	3427	3437	2	2
●	3	dsRNA	Birnaviridae	2	5711	6677	2	3
●	3	dsRNA	Chrysoviridae	4-5	11858	15563	4	5
●	3	dsRNA	Cystoviridae	3	12685	14984	13	19
●	3	dsRNA	Endornaviridae	1	9760	17635	1	1
●	3	dsRNA	Hypoviridae	1	9149	14581	1	2
●	3	dsRNA	Megabirnaviridae	2	16111	16111	4	4
●	3	dsRNA	Partitiviridae	2-4	3064	6105	2	3
●	3	dsRNA	Picobirnaviridae	2	4270	4270	3	3
●	3	dsRNA	Quadriviridae	4	17078	17078	4	4
●	3	dsRNA	Reoviridae	2-20	6725	46740	1	22
●	3	dsRNA	Totiviridae	1-2	2714	11563	1	5

(Continue on next page)

Supplementary Table 1. (Continued)

Class		Group	Viral family	Genomic Segments	Minimum/Maximum Genome Size (bp)		Minimum/Maximum Proteins encoded	
●	4	ssRNA+	Alphaflexiviridae	1-2	5470	13922	1	10
●	4	ssRNA+	Alphatetraviridae	1-2	6625	7982	2	3
●	4	ssRNA+	Alvernaviridae	1	4375	4375	2	2
●	4	ssRNA+	Arteriviridae	1	12704	15717	10	24
●	4	ssRNA+	Astroviridae	1	6119	7722	2	3
●	4	ssRNA+	Barnaviridae	1	4009	4009	4	4
●	4	ssRNA+	Betaflexiviridae	1	6495	9409	2	7
●	4	ssRNA+	Bromoviridae	3	7830	8870	4	6
●	4	ssRNA+	Caliciviridae	1	6434	8513	2	13
●	4	ssRNA+	Carmotetraviridae	1	6155	6155	4	4
●	4	ssRNA+	Closteroviridae	1-3	13071	19296	3	16
●	4	ssRNA+	Coronaviridae	1	25437	31686	5	37
●	4	ssRNA+	Dicistroviridae	1	8026	10436	2	3
●	4	ssRNA+	Flaviviridae	1-2	8568	21991	3	17
●	4	ssRNA+	Gammaflexiviridae	1	6827	6827	2	2
●	4	ssRNA+	Hepeviridae	1	6654	7310	3	7
●	4	ssRNA+	Iflaviridae	1	8832	10985	1	1
●	4	ssRNA+	Leviviridae	1	3405	4276	4	4
●	4	ssRNA+	Luteoviridae	1	5273	6244	5	8
●	4	ssRNA+	Marnaviridae	1	8587	8587	1	1
●	4	ssRNA+	Mesoniviridae	1	19917	20192	6	7
●	4	ssRNA+	Narnaviridae	1	2343	3765	1	1
●	4	ssRNA+	Nodaviridae	2	4294	6281	2	5
●	4	ssRNA+	Permutotetraviridae	1	5698	5698	5	5
●	4	ssRNA+	Picornaviridae	1-2	6580	14730	11	15
●	4	ssRNA+	Potyviridae	1-2	8224	11295	3	13
●	4	ssRNA+	Roniviridae	1	26253	26253	5	5
●	4	ssRNA+	Secoviridae	1-2	9219	15485	1	4
●	4	ssRNA+	Togaviridae	1	9762	11919	2	19
●	4	ssRNA+	Tombusviridae	1-2	3644	5338	4	8
●	4	ssRNA+	Tymoviridae	1	6035	7564	2	4
●	4	ssRNA+	Virgaviridae	1-3	6279	12293	3	9
●	5	ssRNA-	Arenaviridae	2	10056	10719	4	4
●	5	ssRNA-	Bornaviridae	1	8884	8914	6	6
●	5	ssRNA-	Bunyaviridae	3	11372	19146	3	5
●	5	ssRNA-	Filoviridae	1-2	18875	38225	8	14
●	5	ssRNA-	Ophioviridae	1-4	1305	12499	1	7
●	5	ssRNA-	Orthomyxoviridae	6-8	10461	14452	7	12
●	5	ssRNA-	Paramyxoviridae	1	13335	19212	6	12
●	5	ssRNA-	Rhabdoviridae	1	10692	15867	5	15
●	6	ssRNA-RT	Metaviridae	NA	NA	NA	NA	NA
●	6	ssRNA-RT	Retroviridae	1	2630	13246	2	24
●	7	dsDNA-RT	Caulimoviridae	1	6949	9314	1	9
●	7	dsDNA-RT	Hepadnaviridae	1	3018	3377	3	7
